# Comparing the clinical outcomes in stereotactic body radiotherapy for lung tumors between Ray-Tracing and Monte-Carlo algorithms

**DOI:** 10.18632/oncotarget.5992

**Published:** 2015-10-26

**Authors:** Jin Ho Song, Ki Mun Kang, Hoon-Sik Choi, Hojin Jeong, In Bong Ha, Jong Deog Lee, Ho Cheol Kim, Yi Yeong Jeong, Yu Ji Cho, Seung Jun Lee, Sung Hwan Kim, In-Seok Jang, Bae Kwon Jeong

**Affiliations:** ^1^ Department of Radiation Oncology, Gyeongsang National University School of Medicine and Gyeongsang National University Hospital, Jinju, Republic of Korea; ^2^ Department of Internal Medicine, Gyeongsang National University School of Medicine and Gyeongsang National University Hospital, Jinju, Republic of Korea; ^3^ Department of Thoracic and Cardiovascular Surgery, Gyeongsang National University School of Medicine and Gyeongsang National University Hospital, Jinju, Republic of Korea; ^4^ Institute of Health Sciences, Gyeongsang National University, Jinju, Republic of Korea

**Keywords:** stereotactic body radiotherapy, lung cancer, calculation algorithm, CyberKnife, Monte Carlo

## Abstract

**Purpose:**

The purpose of this study was to compare the clinical outcomes between the groups using Ray-Tracing (RAT) and Monte-Carlo (MC) calculation algorithms for stereotactic body radiotherapy (SBRT) of lung tumors.

**Materials and Methods:**

Thirty-five patients received SBRT with CyberKnife for 47 primary or metastatic lung tumors. RAT was used for 22 targets in 12 patients, and MC for 25 targets in 23 patients. Total dose of 48 to 60 Gy was prescribed in 3 to 5 fractions on median 80% isodose line. The response rate, local control rate, and toxicities were compared between RAT and MC groups.

**Results:**

The response rate was lower in the RAT group (77.3%) compared to the MC group (100%) (*p* = 0.008). The response rates showed an association with the mean dose to the gross tumor volume, which the doses were re-calculated with MC algorithm in both groups. However, the local control rate and toxicities did not differ between the groups.

**Conclusions:**

The clinical outcome and toxicity of lung SBRT between the RAT and MC groups were similar except for the response rate when the same apparent doses were prescribed. The lower response rate in the RAT group, however, did not compromise the local control rates. As such, reducing the prescription dose for MC algorithm may be performed but done with caution.

## INTRODUCTION

Stereotactic body radiotherapy (SBRT) is one of the treatment options for early stage non-small cell lung cancer (NSCLC) or metastatic lung lesions [1–9]. SBRT can accurately target and deliver high doses of radiation, and therefore can achieve high local tumor control with fewer complications than conventional radiotherapy (RT). CyberKnife (Accuray Inc., Sunnyvale CA, USA) used in this study is a frameless robotic SBRT system delivering 6-MV photons with no flattening filter, and incorporating an image-guided system which allows near real-time tracking of a moving tumor [3–7].

Since SBRT delivers extremely hypo-fractionated high radiation dose, an exact dose calculation is essential. The issue is more challenging when the tumor is located within or nearby a heterogeneous region, such as lung and air cavity. In heterogeneous region, the calculated dose is more likely to be deviated from the real dose because of increase in transmittance radiations [10–12]. CyberKnife treatment planning system incorporates two different dose calculation algorithms, referred as Ray-Tracing (RAT) and Monte-Carlo (MC) algorithms [12–14].

The two algorithms utilize different correction methods for tissue heterogeneity. The RAT algorithm basically utilizes the beam data set premeasured in a water phantom, and the tissue heterogeneity is corrected only along the propagation direction of the primary photons [12–14]. The beam attenuation is estimated using the radiological effective path length (EPL) instead of the actual physical length. No other corrections for scatted and transmittance radiations are considered in the RAT algorithm. On the other hand, the MC algorithm integrates all kinds of radiation interactions with tissues based on well-established physical theories and stochastic sampling schemes [12–14].

So far, a large number of studies have been reported to validate the calculation accuracies of RAT and MC methods for SBRT of lung cancer, reaching two findings [10–16]: i) the RAT algorithm generally overestimates the dose compared with MC and ii) the MC dose is closer to the real dose than the RAT dose. Because of the latter result, current SBRT dose calculations for lung tumors are rapidly adopting the MC algorithm. However, the former result may be problematic because the MC-based dose prescription may not be consistent with the existing general guidelines that have been established empirically from clinical data based on conventional algorithms such as RAT.

Several studies have shown that the dose differences between RAT and MC calculations amounted to more than 10%, and this difference is not negligible in performing SBRT of lung tumors [13–16]. Thus, the dose prescription must be carefully adjusted from the general recommendation if using the MC algorithms, as suggested by some investigators [17, 18]. However, this may not be simple as there are not sufficient clinical data for an adjustment of an MC-based dose prescription or using an appropriate conversion method for an MC dose to a conventional one.

At our institution, SBRT with CyberKnife application in lung tumors was initially based on RAT algorithm, but it has changed to MC with no additional changes to the treatment protocol. In this study, as an initial attempt to standardize the MC-based dose prescription for heterogeneous lung SBRT, we retrospectively analyzed our clinical data of SBRT in lung tumors and compared the treatment outcome between RAT and MC algorithms. This study was reviewed and approved by the institutional review board (IRB) of the Gyeongsang National University Hospital (IRB No. 2015-07-011).

## RESULTS

### Baseline characteristics of RAT and MC groups

Table 1 compares the patient and tumor characteristics between the RAT and MC groups. There were no statistically significant differences in baseline characteristics between RAT and MC groups, except for the number of treated targets. In the RAT group, 6 (50%) patients received SBRT on multiple targets (three patients for two targets, two patients for three targets, one patient for four targets). In contrast, only two (8.7%) patients received SBRT on multiple targets in the MC group. Other characteristics including patient age, sex, classification, tumor site, location, pathology and tumor size showed no meaningful difference between the groups. The dose prescription policy between the two groups also did not differ.

**Table 1:** Patient and target characteristics

Patient characteristics	Total (*n* = 35) (%)	RAT (*n* = 12) (%)	MC (*n* = 23) (%)	*p* value
Age	Median 71 (range: 51–92)			0.685
≥65 years	26 (74.3)	8 (66.7)	18 (78.3)	
<65 years	9 (25.7)	4 (33.3)	5 (21.7)	
Sex				0.434
Male	25 (71.4)	10 (83.3)	15 (65.2)	
Female	10 (28.6)	2 (16.7)	8 (34.8)	
Classification				0.557
Primary lung cancer	16 (45.7)	4 (33.3)	12 (52.2)	
Recurrent lung cancer	9 (25.7)	4 (33.3)	5 (21.7)	
Metastases	10 (28.6)	4 (33.3)	6 (26.1)	
No. of treated sites				0.011
Single	27 (77.1)	6 (50.0)	21 (91.3)	
Multiple	8 (22.9)	6 (50.0)	2 (8.7)	
ECOG PS				0.402
0 or 1	27 (77.1)	8 (66.7)	19 (82.6)	
2	8 (22.9)	4 (33.3)	4 (17.4)	
**Target characteristics**	Total (*n* = 47) (%)	RAT (*n* = 22) (%)	MC (*n* = 25) (%)	*p* value
Tumor origin				0.539
Lung tumor	32 (68.1)	14 (63.6)	18 (72.0)	
Metastatic tumor	15 (31.9)	8 (36.4)	7 (28.0)	
Sites				0.447
Right upper lobe	12 (25.5)	4 (18.1)	8 (32.0)	
Right middle lobe	3 (6.4)	2 (9.1)	1 (4.0)	
Right lower lobe	10 (21.3)	7 (31.8)	3 (12.0)	
Left upper lobe	11 (23.4)	5 (22.7)	6 (24.0)	
Left lower lobe	11 (23.4)	4 (18.2)	7 (28.0)	
Location				0.144
Central	9 (19.1)	2 (9.1)	7 (28.0)	
Peripheral	38 (80.9)	20 (90.9)	18 (72.0)	
Target size				0.072
≤3 cm	30 (63.8)	17 (77.3)	13 (52.0)	
>3 cm	17 (36.2)	5 (22.7)	12 (48.0)	
Prescription dose				0.345
48–50 Gy	8 (17.0)	2 (9.1)	6 (24.0)	
60 Gy	39 (83.0)	20 (90.9)	19 (76.0)	

*Abbreviations*: RAT, Ray-Tracing group; MC, Monte-Carlo group; ECOS PS, Eastern Cooperative Oncology Group Performance Status;

### Treatment results

Table 2 shows the overall treatment response of all targets and results between the groups. There were 7 (14.9%) targets which showed CR, 35 (74.5%) targets with PR, and 5 (10.6%) targets with SD. All the targets which showed SD both in CT and PET-CT evaluation belonged to RAT group. All 25 targets in the MC group showed at least a PR. The overall response rate was 77.3% in the RAT group, and 100% in the MC group. This difference of target responses was statistically significant (*p* = 0.008).

**Table 2:** Treatment response

Overall response	Total (*n* = 47) (%)	RAT (*n* = 22) (%)	MC (*n* = 25) (%)	*p* value
Complete response	7 (14.9)	5 (22.7)	2 (8.0)	0.008
Partial response	35 (74.5)	12 (54.5)	23 (92.0)	
Stable disease	5 (10.6)	5 (22.7)	0 (0.0)	

*Abbreviations*: RAT, Ray-Tracing group; MC, Monte-Carlo group

**Figure 1:**
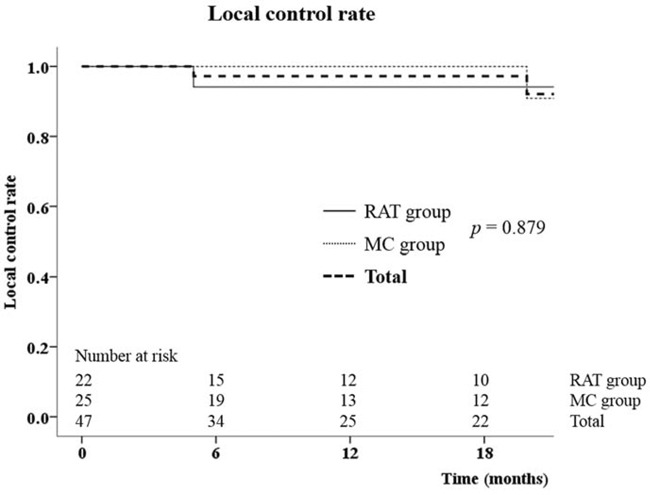
The local control rate between Ray-Tracing (RAT) and Monte-Carlo (MC) groups

Table 3 shows the LCR and survival outcomes. After median 13.2 months (range: 2.4–60.4) follow-up, there were only two targets that showed progression. One target was in the RAT group, and the other was in the MC group. The 2-year LCR was 94.1% in the RAT group, and 90.9% in the MC group with no statistically significant difference (*p* = 0.879) (Figure 1). The DFS and OS were also not different by the calculation algorithms (*p* = 0.408, 0.311). The 2-year DFS and OS for the whole of patients were 29.0% and 60.1%, respectively.

**Table 3:** Prognostic factors affecting the response rate, 2-year local control rate, and survival

	Response rate	*p* value	Local control rate	*p* value	Disease-free survival	*p* value	Overall survival	*p* value
Algorithm		0.008		0.879		0.408		0.311
RAT group	77.3%		94.1%		43.8%		38.9%	
MC group	100.0%		90.9%		21.5%		67.1%	
Tumor origin		0.309		0.367		<0.001		0.088
Lung cancer	93.8%		88.5%		38.2%		62.2%	
Metastasis	80.0%		100.0%		10.0%		50.0%	
RT dose		0.571		0.132				
48–50 Gy	100.0%		83.3%		n/a		n/a	
60 Gy	87.2%		94.1%		n/a		n/a	
Tumor size		0.143		0.629				
≤3 cm	83.3%		95.7%		n/a		n/a	
>3 cm	100.0%		83.3%		n/a		n/a	

*Abbreviations*: RAT, Ray-Tracing group; MC, Monte-Carlo group

### Toxicity results

The most common toxicity was fatigue and anorexia (Table 4). Nineteen patients (54.3%) experienced grade 1 or 2 fatigue and anorexia, while 13 (37.1%) patients complained of chest wall pain. One patient had multiple rib fractures without a trauma history 16 months after from the end of treatment. No grade 3 or higher toxicities were observed except for the lung. There were 10 (28.6%) patients who suffered from ≥ grade 2 inflammation of the lung. Of these 10 patients, 8 patients were diagnosed with radiation pneumonitis in whom the pneumonia was developed within 6 months after the end of treatment and required steroid treatment. Six patients needed admission care for the pneumonia treatment (grade 3), and one patient died 5 months after the SBRT (grade 5). Grade 2 or higher radiation pneumonitis were more frequent in the RAT group (41.7%) compared to the MC group (13.0%) (*p* = 0.091) although the difference did not reach significance. This may have been due to having more patients in the RAT group having been treated for multiple targets and suggested by logistic regression analysis (Odds ratio: 9.8, 95% confidence interval: 1.3–76.5, *p* = 0.030) (Table 5).

**Table 4:** Toxicities

	Total (*n* = 35) (%)	RAT (*n* = 12) (%)	MC (*n* = 23) (%)	*p* value
Lung toxicities (≥ Grade 2)				
Pneumonitis	10 (28.6)	6 (50.0)	4 (17.4)	0.059
Radiation pneumonitis	8 (22.9)	5 (41.7)	3 (13.0)	0.091
Other pneumonia	2 (5.7)	1 (8.3)	1 (4.3)	1.000
Hemoptysis	2 (5.7)	1 (8.3)	1 (4.3)	1.000
Other toxicities (Grade 1–2)*				
Fatigue or anorexia	19 (54.3)	8 (66.7)	11 (47.8)	0.476
Chest wall pain^†^	13 (37.1)*	6 (50.0)	7 (30.4)^†^	0.292
Dermatitis	4 (11.4)	1 (8.3)	3 (13.0)	1.000

*Abbreviations*: RAT, Ray-Tracing group; MC, Monte-Carlo group

* No grade 3 or higher toxicities were observed.

† Including one rib fracture

**Table 5:** Factors affecting grade 2 or higher radiation pneumonitis

Factors	Radiation pneumonitis (*n* = 8)	Univariate analysis (p)	Adjusted odds ratio (95% confidence interval)	Multivariate analysis (p)
Algorithm RAT group MC group	5/12 (41.7%) 3/23 (13.0%)	0.091	0.5 (0.1–3.9)	0.522
Treated targets Single Multiple	3/27 (11.1%) 5/8 (62.5%)	0.007	9.8 (1.3–76.5)	0.030
RT dose 48–50 Gy 60 Gy	0/5 (0.0%) 8/30 (26.7%)	0.315	n/a	
Target size ≤3 cm >3 cm	5/19 (26.3%) 3/16 (18.8%)	0.700	n/a	

### Dosimetric comparison

Table 6 shows the dosimetric comparison of the targets. The GTV and PTV mean doses were 120.2 ± 3.9% and 110.4 ± 5.1%, respectively, of the prescription dose in the RAT group by the calculation of RAT algorithm. However, after recalculating the RAT plans with the MC algorithm (re-MC plans), the mean doses of both GTV and PTV decreased in a large extent of 12.6% and 18.0%, respectively (*p* < 0.001). The minimum doses of GTV and PTV also decreased by 23.5% and 18.6% after recalculating the RAT plans with MC algorithm (*p* < 0.001).

**Table 6:** Dosimetric comparison

Groups	GTV mean	PTV mean	GTV min	PTV min
RAT plans	120.2 ± 3.9	110.4 ± 5.1	110.2 ± 7.9	73.1 ± 12.4
*p* value	<0.001	<0.001	<0.001	<0.001
Re-MC plans	107.6 ± 7.9	92.4 ± 9.9	86.7 ± 11.4	54.5 ± 8.7
*p* value	<0.001	<0.001	0.154	<0.001
MC plans	120.6 ± 7.7	112.8 ± 7.4	91.8 ± 11.8	74.9 ± 11.8
**Overall response**				
Responding	116.0 ± 9.6	105.1 ± 12.9	89.5 ± 11.6	65.5 ± 16.5
*p* value	0.023	0.056	0.985	0.462
Non-responding	104.1 ± 10.3	91.8 ± 11.2	89.7 ± 15.3	59.2 ± 10.5

*Abbreviations*: RAT, Ray-Tracing group; MC, Monte-Carlo group; Re-MC, recalculating RAT plans with MC algorithm; CR, complete response; PR, partial response; SD, stable disease, GTV, gross tumor volume; PTV, planning target volume

If we compared the re-MC plans to the plans of MC group, the GTV and PTV mean doses were significantly lower in the re-MC plans (*p* < 0.001). The magnitude of difference was 13.0% for the GTV, and 20.4% for the PTV. The minimum dose of PTV was also lower in the re-MC plans (54.5% vs. 74.9%, *p* < 0.001), while the difference of GTV minimum dose did not reach statistical significance (86.7% vs. 91.8%, *p* = 0.154).

We also compared the minimum and mean doses for the targets between responding group and non-responding group (Table 5). For this comparison, only the doses calculated by the MC algorithm were used for a consistent comparison. The GTV mean dose was 11.9% higher in the responding group than the non-responding group (*p* = 0.023). The PTV mean dose also differed between responding groups and non-responding group with marginal significance; however, the minimum doses of GTV and PTV was similar.

## DISCUSSION

There are several reports that showed the dose difference between the calculation algorithms [12–16]. Xiao et al. analyzed a subset of 20 patients participating in the RTOG 0236 study [15]. They recalculated the plans with a heterogeneity corrections algorithm, and showed that the PTV V_60Gy_ decreased on average by 10.1%. Van der Voort van Zyp et al. also analyzed 53 SBRT CyberKnife treatment plans and compared the dosimetric results between RAT and MC [13]. They showed that the PTV D_95%_ and D_99%_ decreased from 10 to 21% according to tumor size when the RAT plans were re-calculated with MC. Therefore they concluded the prescription dose in the MC calculated plans should be 10–20% lower (16 to 18 Gy per fraction) than the RAT plans (20 Gy per fraction) to deliver effectively the same dose. Wu et al. also compared the RAT plans and re-calculated MC plans [16]. They showed the PTV D_95%_ decreased from 50.0 Gy in RAT plans to 42.9 Gy in the MC plans (14.2% decrease), and this deviation tended to be greater in small peripheral targets than the large central targets. All of these dosimetric results showed that the actually delivered dose to the target was 10 to 20% lower in the RAT plans, and suggested to deliver actually same dose to the target, the prescription dose should be decreased when using MC algorithms. Therefore, recent clinical trials, such as the STARTS and ROSEL, adopted this dose scheme, and recommend 54 Gy in 3 fractions for peripheral tumors when ‘heterogeneity correction’ algorithms are used [17, 18].

Nevertheless, most clinical data have used similar prescription doses regardless which algorithms were used (such as 60 Gy in 3 fractions for peripheral tumors), and reported similar LCR and toxicities [1, 6, 19–21]. In addition, Latifi et al. reported inferior local control rates (with hazard ratio of 3.4) in patients treated with pencil-beam algorithm compared to collapsed cone convolution when 50 Gy in 5 fractions were prescribed [22]. Therefore, reducing the prescription dose in MC algorithm may imply a risk of tumor recurrence, and therefore is not a clear answer.

In our study, we also showed that the GTV and PTV mean dose was 10 to 20% higher in the MC group. Therefore, we expected the tumor control rate will be higher in the MC group, or that the toxicity will be higher in the MC group with similar tumor control rate.

However, the difference was shown only in the response rate. The response rate was higher in the MC group even though the clinical characteristics were similar. The difference in response rate seems to be due to the difference in delivered doses to the target. The GTV mean doses were higher in the responding targets compared to non-responding targets when evaluating based on MC-recalculated dose. Despite the differing responding rate, the local-control rate was similar for RAT and MC, and both were over 90%. Most of the tumors classified as SD did not progress during the follow up, and the doses actually delivered in the RAT groups were sufficient to acquire local control. The LCR in our study were comparable with those in other lung SBRT studies [1–7]. The development of radiation pneumonitis did not differ between RAT and MC groups, and correlated with the number of treated targets (single versus multiple).

From our data on LCR and toxicity, the strategy of reducing the prescription dose in 10 to 20% for MC calculation seems reasonable. However, our results also suggest that this strategy should be performed with caution since there can be a risk of decreased tumor response, even though this did not compromise local control rates.

Our study had some limitations such as its retrospective nature and the small number of patients analyzed. A longer follow-up time could have also benefited the analysis. Also the tumor response was collected with at a rather large time window (range 1.2 to 6.9 months post treatment), and the metabolic response data was only evaluable in half of the patients. Nevertheless our study is remarkable since to our knowledge, no other study exists which compares the RAT and MC algorithms with respect to clinical outcome. In addition, since all baseline characteristics as well as the treatment protocols were similar between groups except for the calculation algorithm, our result seems reliable in comparing the clinical results between RAT and MC algorithms.

In conclusion, the clinical outcome and toxicity of lung SBRT between the RAT and MC groups were similar except for the response rates seen, when apparently the same but in reality not the same doses were prescribed. The lower response rate, however, did not compromise the local control and survival. Therefore we recommend reducing the prescription dose for MC algorithm should be performed with caution.

## MATERIALS AND METHODS

### Patient and tumor characteristics

From March 2010 to July 2014, 35 patients were treated with SBRT for 47 lung tumors at Gyeongsang National University Hospital. The patient characteristics are summarized in Table 1. There were 25 male and 10 female patients with median age of 71 years (range: 51–92 years). The Eastern Cooperative Oncology Group (ECOG) performance statuses were rated 0 or 1 for 27 patients (77.1%), and rated 2 for eight patients (22.9%). Twenty-seven (77.1%) patients were treated for single lesion, and eight patients (22.9%) for multiple lesions (range: 2–4 lesions).

There were 16 patients (17 lesions) who were diagnosed as primary NSCLC and were determined to received SBRT either because of inoperability or patient refusal of surgery. Nine patients (15 lesions) received SBRT because of recurrent NSCLC. These patients had previously been treated with surgery or concurrent chemoradiotherapy for primary lung cancers, and the developed recurrences were only in the lungs. Six patients were treated for single lesion, three patients for multiple lesions (2–4 lesions). Ten patients (15 lesions) received SBRT for lung metastases from other primary sites which were in controlled state.

Of 47 lesions, 32 (68.1%) lesions were NSCLC and 15 (31.9%) lesions were metastatic tumors. The metastatic tumors came from hepatocellular carcinoma for 5 lesions, renal cell carcinoma for 3 lesions, and esophageal cancer for 3 lesions. Other 4 lesions were stomach cancer, pancreas cancer, rectal cancer, and sarcoma, respectively. There were 38 (80.9%) peripheral tumors, and 9 (19.1%) central tumors on the basis of the relative tumor position from the proximal bronchial tree by the definition of Radiation Therapy Oncology Group (RTOG) 0236 study [20]. The tumor sizes were 3 cm or smaller in 30 (63.8%) lesions and larger than 3 cm in 17 (36.2%) lesions with median size of 2.3 cm (range: 0.7 to 4.8 cm).

### Radiotherapy technique

In all patients, two or three gold seed fiducials were inserted around the target, and one week after, planning computed-tomography (CT) was taken with 1.25 mm slice thickness. The gross tumor volume (GTV) was outlined on the planning CT image in lung window setting, the clinical target volume (CTV) was the same as the GTV, and a volumetric margin of 3 mm was applied to make the planning target volume (PTV). The tumor dose was prescribed on the median 80% (range: 68–84%) isodose line with respect to the global maximal dose point which covers 95% volume of PTV. The prescription dose was 60 Gy in 3 or 4 fractions for peripheral tumors (39 targets), 48 Gy in 4 fractions for central tumors (7 targets), and 50 Gy in 5 fractions for one central tumor abutting the heart.

The treatment planning was performed with the CyberKnife planning system (MultiPlan, Accuray Inc., Sunnyvale CA, USA). Before applying the MC calculation algorithm in August 2010, the dose was calculated and optimized with the RAT algorithm for 12 patients and 22 targets (RAT group). Since then, the dose is calculated with the MC algorithm for 23 patients and 25 targets (MC group). All patients were treated once a day for 3 to 5 days with a real-time tumor tracking system called the Synchrony Respiratory Tracking System (Accuray).

Before treatment, we obtained a dynamic respiratory rhythm via a respiratory monitoring device that detects the position of the infrared generator placed on the chest. Two orthogonal kV X-ray images were obtained at different times during the respiratory cycle. The dynamic model between the fiducial position and respiratory rhythm was used as a guide to tracking the lesions and delivering dynamic radiation.

### Treatment outcome evaluation

The endpoint in our study was response rate and local control of the irradiated target. For the response evaluation, we compared the planning CT and the first follow-up CT, which was acquired at median of 2.2 months (range: 1.2 to 2.7 months) after the end of SBRT in the RAT group, and at median of 2.7 months (range: 1.2 to 6.9 months) in the MC group. We adopted the first follow-up CT for comparison since in the later CTs the radiation-related changes in lung would make it difficult to evaluate the response precisely. The Response Evaluation Criteria in Solid Tumors (RECIST) version 1.1 was used, which defined complete response (CR) as disappearance of target lesions, partial response (PR) as >30% decrease of the longest diameter of the target, progressive disease (PD) as >20% increase of the longest diameter of target, and stable disease (SD) as targets which are neither sufficient for PR or PD criteria [23]. Additionally, pre- and post-SBRT positron-emission tomography (PET)-CT were available for 29 (61.7%) targets and for these targets, metabolic tumor responses were also evaluated by the PET Response Criteria in Solid Tumors (PERCIST) [24]. If both CT and metabolic responses showed a complete response (CR), then the overall response was regarded as a CR. If both responses showed stable disease (SD), then the overall response was regarded as SD. If CT or metabolic response showed different results, the overall response was chosen from the worse response. If only CT response was evaluable then the CT response result was regarded as the overall response. There was no progressive disease neither in CT and metabolic response evaluation in the first follow-up. We defined targets that had shown CR and PR as ‘responding’ group, and targets with SD as ‘non-responding’ group.

Local control rate (LCR) was defined as the absence of tumor re-growth after initial shrinkage. Although it is sometime difficult to distinguish tumor regrowth from radiation-related lung injury, tumor recurrence was considered if a solid homogeneous mass increasing in size during follow-up was evident. The disease-free survival (DFS), and overall survival (OS) were also analyzed along with the prognostic factors. The time was calculated from the end of SBRT to development of the event, and the event was defined as follow: i) LCR event was any progression of the treated target, ii) DFS event was any progression or recurrence of the primary or metastatic tumor, and iii) OS event was death from any cause. Toxicity was evaluated by National Cancer Institute's Common Terminology Criteria for Adverse Events (CTCAE) version 4.0. [25].

### Dosimetric comparison

We compared the dosimetric data between the RAT and MC groups. With our reliance on MC algorithm as the ‘gold standard’, we recalculated the RAT plans with the MC algorithm while keeping the patient data and the dosimetric parameters such as the beam numbers, directions, and monitor units constant. The changes in the mean and minimum doses of GTV and PTV by the MC recalculation were then evaluated. We also compared the recalculated MC doses in the RAT group to those in the MC group.

To find the correlation between the dosimetric data and clinical outcome, the mean and minimum doses of GTV and PTV by the MC recalculation were compared between the ‘responding’ and ‘non-responding’ groups.

### Statistical analysis

To compare the patient and tumor characteristics, as well as the toxicities between RAT group and MC groups, Chi-square test and Fisher's exact test were used. The LCR, DFS, and OS were evaluated by the Kaplan-Meier method and were compared by log-rank test. To find the clinical factors affecting lung toxicity, multivariate logistic regression analysis was performed with backward stepwise elimination method. To compare the dosimetric data between the groups student *t*-test was used. All statistical analysis was performed by using SPSS version 21.0 (Chicago, IL, USA) and was considered significant with a *p* value <0.05 by two-tailed tests.
